# A Novel Forecasting Approach by the GA-SVR-GRNN Hybrid Deep Learning Algorithm for Oil Future Prices

**DOI:** 10.1155/2022/4952215

**Published:** 2022-08-21

**Authors:** Liang Wang, Yang Xia, Yichun Lu

**Affiliations:** ^1^College of Business, Shanghai University of Finance and Economics, Shanghai 200433, China; ^2^School of International Business, Guangxi University, Nanning 530004, China

## Abstract

It is hard to forecasting oil future prices accurately, which is affected by some nonlinear, nonstationary, and other chaotic characteristics. Then, a novel GA-SVR-GRNN hybrid deep learning algorithm is put forward for forecasting oil future price. First, a genetic algorithm (GA) is employed for optimizing parameters regarding the support vector regression machine (SVR), and the GA-SVR model is used to forecast oil future price. Further, a generalized regression neural network (GRNN) model is built for the residual series for forecasting. Finally, we obtain the predicted values of the oil future price series forecasted by the GA-SVR-GRNN hybrid deep learning algorithm. According to the simulation, the GA-SVR-GRNN hybrid deep learning algorithm achieves lower MSE, RMSE, MAE, and MAPE relative to the GRNN, GA-SVR, and PSO-SVR models, indicating that the proposed GA-SVR-GRNN hybrid deep learning algorithm can fully reveal the prediction advantages of the GA-SVR and GRNN models in the nonlinear space and is a more accurate and effective method for oil future price forecasting.

## 1. Introduction

Oil, as a strategic resource, is presently playing a critical role in the economic and social operations of countries all over the world. However, it is hard to forecasting oil future prices accurately, which is affected by some nonlinear, nonstationary, and other chaotic characteristics [1]. Until now, researchers have not reached a consensus on the technologies and models used for oil future price prediction, and large number of researchers have done work to help solve this problem [2–8]. The oil future price forecasting has always been an important and challenging issue and widely studied by scholars. Many researchers have found that oil future price has chaotic features such as nonstationary and nonlinear. These features usually bring thorny challenges to researchers who aim to forecast oil future price [9]. Therefore, employing some related deep learning algorithms or statistical models into oil future price prediction is still a popular and interesting topic in growing literature [10–18]. Existing research proposes methods for predicting oil future price from various angles, which can basically be summarized as four aspects to enhance the accuracy of prediction: (1) statistical model, (2) deep learning algorithm, (3) hybrid methodology, and (4) parameter optimization.

On the one hand, many local and international academics have focused on the oil future price forecasting using statistical models in the past, mostly using linear statistical models for enhancing the forecasting accuracy of oil future price. Autoregressive integrated moving average (ARIMA) model for the forecasting of oil future price was adopted by many literature [10–14]. Later, the hidden Markov model (HMM), dynamic model averaging (DMA), and ARCH approach were adopted by many researchers for forecasting oil future price [15–17]. Previous scholars have made some progress and breakthroughs in forecasting oil future prices using the statistical method. However, considering the inherent nonlinear and chaotic characteristics existed in oil future price, the statistical method is not powerful enough for predicting the oil future price. On the other hand, as deep learning technology develops, more and more literature using deep learning algorithms to forecast oil future price in current research. Benefiting from the in-depth development of deep learning, deep learning methods are more and more widely used for predicting oil future price. The support vector regression machine (SVR) is a famous deep learning algorithm for oil future price prediction, which widely employed for forecasting the oil future price [17, 18]. Yu et al., for example, build an LSSVR ensemble learning paradigm for the oil future price forecasting by treating user-defined parameters as uncertain (or random) components [19]. In machine learning field, SVR presents the advantage that it can effectively solve nonlinear problems, and overfitted problems, which is better than some other deep learning algorithm such as artificial neural network (ANN) [1, 20–23].

Simultaneously, due to the aim of increasing model prediction accuracy and addressing the drawbacks of single model, more and more hybrid models were employed for forecasting oil future price [24–27]. On the one hand, SVR is often adopted into hybrid model for enhancing the accuracy of oil future price [27–31].On the other hand, GRNN model was also frequently employed in hybrid model for effectiveness of forecasting [32, 33]. Concerning the different merits of SVR and GRNN, it is necessary to incorporate SVR and GRNN for enhancing the prediction accuracy. Furthermore, researchers refined the nonparametric prediction model from the standpoint of parameter optimization and mostly used genetic algorithm (GA) to optimize the results [34–36]. GA method have proved that it is necessary to optimize model's parameter by it.

The effectiveness of “deep learning algorithm,” “hybrid model,” and “parameter optimization” has been proved by previous literature. In view of the potential of SVR, GRNN, and GA in the parameter optimization, respectively, we developed a GA-SVR-GRNN hybrid deep learning algorithm for forecasting oil future price. First, a GA is used for optimizing the parameters of SVR, and the GA-SVR model employed to forecast oil future price. Further, a GRNN model is constructed for forecasting residual series generated by GA-SVR. At last, the predicted values of the GA-SVR-GRNN hybrid model are obtained for the oil future price.

Our marginal contributions can be summed up as follows:

First, a novel hybrid deep learning algorithm that correctly incorporated SVR, GRNN, and GA is suggested. To our knowledge, this is the first time the GA-SVR-GRNN hybrid model has been used for predicting oil future price, and it fully utilizes the SVR and GRNN strengths of deep learning algorithms. Second, GA is employed to optimize parameters of SVR, which aim to improve predicting performance even more. Third, results of experiments reveal that the GA-SVR-GRNN hybrid deep learning algorithm achieves lower MSE, RMSE, MAE, and MAPE relative to the GRNN, GA-SVR, and PSO-SVR models.

The key novelty of the study also can be summed up as follows:

First, a novel ensemble model incorporating the three methods is developed for forecasting oil future price, inspired by the effective combination of SVR, the powerful optimization ability of GA, and the potential prediction performance of GRNN. Second, GA-SVR and GRNN are first combined, and GA is applied to optimizing parameter simultaneously. Third, the proposed GA-SVR-GRNN hybrid deep learning algorithm is first developed to predict the oil future price, and the experiment results verify the effectiveness of the model.

In the following sections, we propose the concept and algorithm of the GA-SVR-GRNN hybrid deep learning algorithm and provides a short introduction to GA, SVR, and GRNN in Section 2. The benchmark experimental results on the forecasting of oil future price by the GA-SVR-GRNN hybrid deep learning algorithm are presented in Section 3.  Section 4 reports and discusses the additional experimental results on oil future price forecasting by comparative model, including GRNN, GA-SVR, and PSO-SVR models. Finally, Section 5 gives concluding remarks and policy implication.

## 2. Materials and Methods

### 2.1. GA-SVR-GRNN Process Description

1. Obtain and input oil future price time series
2. Utilize GA and use MSE as fitness function (objective function) to perform five-fold interactive verification on the sequences above, respectively, in order to find the optimal penalty parameter C, the insensitive loss function *ε* as well as the RBF kernel function parameters *γ* of SVR corresponding to the weekly oil future price time series
3. Based on above indicators of *C*, *ε*, and *γ* of three fuzzy information granular sequences, further divide it into the training set and the test set meanwhile establishing GA-SVR model for the training and the prediction
4. Output the generated residual sequence corresponds to the training of the GA-SVR model, followed by using the GRNN model for correcting the prediction result from GA-SVR training residual
5. Establish GA-SVR-GRNN hybrid deep learning algorithm and output the forecast results regarding oil future price time series

**Figure 1:**
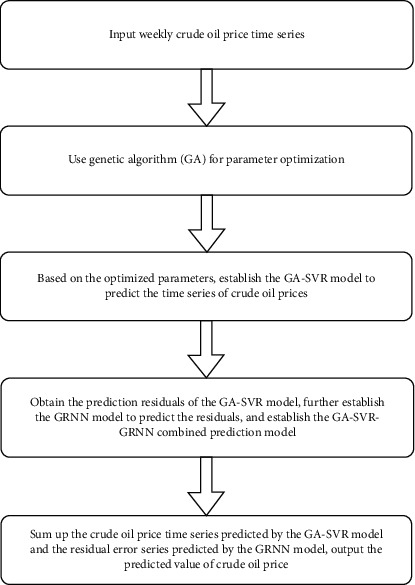
GA-SVR-GRNN hybrid deep learning algorithm flow chart.

All in all, SVR has excellent generalization ability; shows distinguished advantage in solving small sample; nonlinear as well as high-dimensional spaces; and can avoid dimensional disasters, local extreme values, and other problems. As show in Figure 1, the outstanding feature of GA is easy to find the global optimal solution with high computational efficiency and can use probabilistic search technology with better self-organization, self-adaptation, and other characteristics, thus genetic algorithm is a simple method with strong versatility. Meanwhile, GRNN model has strong predictive ability for nonlinear time series. Hence, the GA-SVR-GRNN hybrid deep learning algorithm is constructed combining the advantages of the forementioned model.

### 2.2. Genetic Algorithm (GA)

GA, first put forward by Goldberg and Holland, combines Darwin's theory of evolution [37] and then becomes an important optimized algorithm that was adopted by many researches [38–40]. In this work, we use GA to find the optimal penalty parameter *C*, the insensitive loss function *ε*, and the radial basis function (RBF) kernel parameters *γ* in the SVR model, followed by establishing a GA-SVR model for predicting time series of oil future prices. The following are the specific steps:

(1) Select an encoding strategy and specify the values of genetic parameters such as population size, selection, crossover, mutation method, crossover probability *P*_*c*_, and mutation probability *P*_*m*_. As GA uses individual fitness values to evaluate the pros and cons of an individual and determine the size of their genetic opportunities, the evolutionary algebra is set to 200 generations, the population size is set to 20, and the fitness function to MSE (mean squared error). This is the MSE generated by the validation subset from the cross-validation (CV) mechanism capable of effectively measuring the pros and cons of chromosomes in the regression prediction problems as well as avoiding or reducing the phenomenon of overfitting after CV. In this work, we adopted a five-fold CV process with the following fitness function formula:
  (1) $$\begin{array}{c} \text{MSE}=\frac{1}{n}\sum_{i=1}^{n}{\left(y_{p}-y_{p}^{*}\right)}^{2}. \end{array}$$
Here, *y*_*p*_ and *y*_*p*_^*∗*^ denote the observed value and the predicted value, respectively, and *n* is the training set sample size for the fuzzy information particle. Smaller fitness value means better individual effect and greater probability of being selected.
(2) According to the feature subset encoding of each chromosome, complete the encoding operation and generate the initial population *P* randomly. Generally, the nature of the issue to be solved decides which coding scheme shall be selected. Common coding schemes include the binary coding and the real number coding, with the former more commonly used.
(3) Take into account the fitness function for calculating each individual's fitness value. Perform genetic operations using selection, crossover, and mutation operators for the formation of the next generation of populations.
(4) Estimate whether the fitness value satisfies the preset standard; if not, then come back to the previous step or the step 2 for continuing the optimization algorithm until reaching the termination condition. The individual which has the smallest fitness during the evolution is finally confirmed as the optimal individual.

SVR models are good at classifying and regressing, but their optimal generalization performance depends greatly on the setting of parameters. For a given dataset, the most important task is to find the optimal parameters. In practical applications, there is still no proper approach to select parameters during the practical application. At present, parameters can be primarily selected through experiments or a time-consuming CV grid search method.

As a search algorithm with strong robustness that can be used for optimization of complex systems, GA has unique advantages compared with other intelligent algorithms for optimization. GA can easily find the global optimal solution. For GA, the genetic operations are simple, and adopting natural selection with survival of the fittest, *t* is not restricted by conditions of the search space during calculation, and no other auxiliary information is required.

### 2.3. Support Vector Regression (SVR) Machine

The essence of the SVR is the support vector machine that is a neural network model put forward by Vapnik et al. in the 1990s for the investigation of small samples as well as small-probability events [41]. Then, it was applied in the regression prediction field and in many research fields [40]. SVR relies on the structural risk minimization principle for regression estimation, of which the estimation relies on the insensitive loss function *ε*. In addition, SVR uses a risk function that is a combination of penalty terms derived from the principle of minimizing the empirical error and the structural risk. The proposed nonlinear *ε*-SVR is constructed as follows.

Consider a set of data *G*={(*x*_*i*_, *y*_*i*_)}_*i*_^*n*^, thereinto *x*_*i*_ denotes the input feature vector, *y*_*i*_ denotes the target value, and *n* stands for the sample size regarding the time series data. Nonlinear SVR holds a basic idea of mapping data *x* to a high-dimensional feature space by using a nonlinear mapping Φ where linear regression is conducted:

(2) $$\begin{array}{c} f\left(x\right)=\omega^{T}\Phi \left(x\right)+b, \end{array}$$

(3) $$\begin{array}{c} \Phi :R^{n}⟶F,\;\omega \in F. \end{array}$$

In (2) and (3), *b* denotes the threshold value; Φ stands for the high-dimensional feature space, the nonlinear image of the input space *x*. We need to estimate *ω* and *b* to solve the optimization problem; the result can be given by the following equation's minimum value:

(4) $$\begin{array}{c} \frac{1}{2}{\left\|w\right\|}^{2}+C\sum_{i=1}^{i}\left(\xi_{i}+\xi_{i}^{*}\right),\\ s.t\left\{\begin{array}{c} y_{i}-w\cdot \varphi \left(x\right)-b\leq \varepsilon +\xi_{i},\\ w\cdot \varphi \left(x\right)+b-y_{i}\leq \varepsilon +\xi_{i}^{*},\\ \xi ,\xi_{i}^{*}\geq 0,\;i=1,\ldots ,n. \end{array}\right. \end{array}$$

In formula (4), *C* represents the penalty parameter, *ξ*, *ξ*_*i*_^*∗*^ represents the slack variable, and *ε* represents the insensitive loss function. The introduction of *ε* improves the robustness of the estimation. When conducting empirical research, we need to select the parameters *C* and *ε*. Dual theory is generally used to solve the problems above by transforming them into a problem about convex quadratic programming. The Lagrange transformation of (4) can be obtained as follows:

(5) $$\begin{array}{c} L=\frac{1}{2}{\left\|\omega \right\|}^{2}+C\sum_{i=1}^{i}\left(\xi_{i}+\xi_{i}^{*}\right)-\sum_{i=1}^{i}\lambda_{i}\left(\varepsilon +\xi_{i}-y_{i}+\left(\omega ,x_{i}\right)+b\right)-\sum_{i=1}^{i}\lambda_{i}^{*}\left(\varepsilon +\xi_{i}^{*}+y_{i}-\left(\omega ,x_{i}\right)-b\right)-\sum_{i=1}^{i}\left(\eta_{i}\xi_{i}+\eta_{i}^{*}\xi_{i}^{*}\right). \end{array}$$

In formula (5), *λ*_*i*_, *λ*_*i*_^*∗*^, *η*_*i*_, *η*_*i*_^*∗*^ ≥ 0,  *i*=1,…, *n*; the partial derivative of the Lagrangian function regarding the variable *ω*, *b*, *ξ*_*i*_, *ξ*_*i*_^*∗*^ is 0. Importing the Lagrangian operator and optimization restriction formula, formula (5) has the decision function of

(6) $$\begin{array}{c} f\left(x\right)=\sum_{i=1}^{l}\left(\lambda_{i}-\lambda_{i}^{*}\right)k\left(x,x_{i}\right)+b. \end{array}$$

In formula (6), *k*(*x*, *x*_*i*_) denotes the kernel function of SVR. SVR can use the kernel function for mapping the low-dimensional nonlinear raw data to high-dimensional feature space when dealing with nonlinear problems, followed by linear processing in high-dimensional space. Conventional kernel functions are linear kernel functions, polynomial kernel functions, and the Gaussian RBF kernel. Previous research experience indicates that RBF has the best effect when the sample data lack prior knowledge [39]. In this paper, we use RBF as the kernel function in the following form:

(7) $$\begin{array}{c} k\left(x,x_{i}\right)=\text{exp}\left(-\gamma {\left\|x-x_{i}\right\|}^{2}\right),\;\gamma >0. \end{array}$$

The kernel parameter in formula (7) is *γ*. The selection of the *γ* value has an important influence on the kernel function. If it is set too large, it will cause overfitting; if it is set too small, it will weaken the generalization ability of the model.

### 2.4. Generalized Regression Neural Network Model (GRNN)

GRNN was first put forward by Specht [42]. We conduct the GRNN model based on the MATLAB toolbox.

(8) $$\begin{array}{c} {\text{dist}}_{j}=\sqrt{\sum_{i=1}^{R}{\left(x_{i}-IW_{ji}\right)}^{2}},\;\left(j=1,2,\ldots ,M\right). \end{array}$$

The product function of network, netprod, multiplies the elements regarding the hidden layer threshold *b*_1_ and the output elements regarding ‖dist‖ to form the net input *n*^1^, which is passed to transfer function of radbas. *X* and *Y* stand for the input and output of the network, respectively, and *M* is the total training sample number. The first layer is a radial basis hidden layer, and the unit number is the training sample number *M*. dist represents the weight function of modified layer, for determining the distance of the network input from the first layer's weight *IW*_11_:

Generally, its transfer function is the Gaussian function, which is given by

(9) $$\begin{array}{c} a_{j}^{1}=\text{radbas}\left(\text{netprod}\left({\text{dist}}_{j}b_{1j}\right)\right),\\ =\text{exp}\left|-\frac{{\left(n_{j}^{1}\right)}^{2}}{2\sigma_{j}^{2}}\right|,\\ =\text{exp}\left|-\frac{{\left({\text{dist}}_{j}b_{1j}\right)}^{2}}{2\sigma_{j}^{2}}\right|\;\left(j=1,2,\ldots ,M\right). \end{array}$$

In formula (9), *σ*_*j*_ is called the smoothing factor, determining the basis function shape at the position of the *j*th hidden layer. Larger *σ*_*j*_ value means smoother basis function. The linear output layer constitutes its second layer and has a normalized point weight product function nprod. The dot product regarding previous layer output together with the weight *IW*_21_ of this layer constitutes the weight input, which can be directly transformed to transfer function of purlin; the following formula is the network output:

(10) $$\begin{array}{c} y_{k}=\sum IW_{ki}a_{i}^{1}\;\left(k=1,2,\ldots ,s\right). \end{array}$$

Notably, the smoothing factor *σ* remarkably affects the prediction performance exhibited by the network, so selecting a suitable smoothing factor can help to enhance the prediction accuracy of the network. Here, we use the method for determining the smoothing factor proposed and optimized by Specht [20]. Finally, a one-dimensional optimization method is used for the optimization of the smoothing factor *σ* by four steps:

(1) An initial smoothing factor *σ* is set.
(2) A sample is selected from the training sample for testing and the network is built from the remaining samples.
(3) The constructed network model is adopted for the calculation of the absolute value of the error of the test sample, which is called the test error.
(4) Steps 1 and 3 will be repeated till all training samples are used for testing once, followed by finding the average square error between the predicted value of all the points to be estimated and the sample's measured value in this process, that is,
  (11) $$\begin{array}{c} \text{MSE}\left(\sigma \right)=\frac{1}{n}\sum_{i=1}^{n}{\left[{\hat{y}}_{t}-y_{t}\right]}^{2}. \end{array}$$

In formula (11), *y*_*t*_ denotes the training sample value. ${\hat{y}}_{t}$ represents the predicted value of the network following training. The initial smoothing factor is set as 0.1. The smoothing factor sees the optimal value in the case that the above mean square error is the smallest.

## 3. Results and Discussion

### 3.1. Experimental Dataset Source and Evaluation Criteria

The market share of Brent oil occupies over two-third of the world's oil futures trading volume, and it is imported for international market [43]. This paper selects weekly data on North Sea Brent (Brent) oil future settlement prices from June 2, 2017, to May 21, 2021. We followed the above literature and obtained the related data from the WIND database [43]. We selected the first 80% of the total sample observations as a training dataset and choose the remaining 20% of the total sample observations as the test dataset.

Four indicators are employed in the paper for evaluating the experimental results, including the mean square error (MSE), the root mean square error (RMSE), the mean absolute error (MAE), and the mean absolute percentage error (MAPE) [30].

(12) $$\begin{array}{c} \text{MSE}=\frac{\sum_{i}^{N}{\left(P_{i}-O_{i}\right)}^{2}}{N},\\ \text{RMSE}=\sqrt{\frac{\sum_{i}^{N}{\left(P_{i}-O_{i}\right)}^{2}}{N},}\\ \text{MAE}=\frac{\sum_{i}^{N}\left|P_{i}-O_{i}\right|}{N},\\ \text{MAPE}=\frac{\sum_{i}^{N}\left|\left(\left(P_{i}-O_{i}\right)/O_{i}\right)\right|}{N}. \end{array}$$

Here, *N* is the size of the sample, and *O*_*i*_ and *P*_*i*_ represent the real and forecasted results at time *i*, separately. The experiment includes two parts, with the first part using the a forementioned GA-SVR-GRNN hybrid deep learning algorithm for oil future price time-series prediction, and the second part comparing the proposed method with a variety of other methods for verifying whether the GA-SVR-GRNN prediction method exhibits better accuracy and adaptability. This experiment uses the LIBSVM toolkit to complete the corresponding test in the Matlab R2018b environment.

### 3.2. Genetic Algorithm (GA) Optimization Parameters

**Figure 2:**
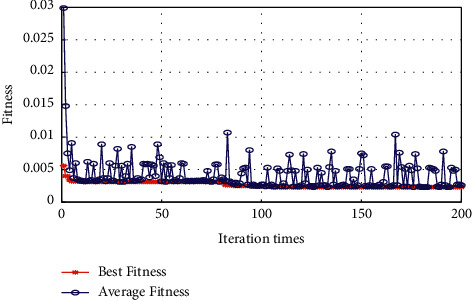
GA optimization parameter fitness curve MSE change graph.

In the environment of Matlab R2018a, this paper uses GA for optimizing the penalty parameter C, the insensitive loss function *ε*, and the RBF kernel function parameters *γ* in the SVR model. We set the evolutionary algebra set to 200 generations, and the population size to 20. The fitness function is selected as the MSE for five-fold cross-validation. The results of the GA process of optimizing parameters are shown in Figure 2. Finally, based on the GA, the optimal penalty parameter *C* determined is 32.6251, the parameter *γ* of the RBF kernel function is 2.7166, and the parameter *ε* of the insensitive loss function is 0.0441.

From Figure 2, we found that the MSE value corresponding to the final optimization result regarding the penalty parameter *C*, the insensitive loss function parameter *ε*, and the parameter *γ* of the RBF kernel function are all less than 0.005, indicating the optimization parameter result of the genetic algorithm (GA) has strong adaptability.

### 3.3. GA-SVR Model Prediction

**Figure 3:**
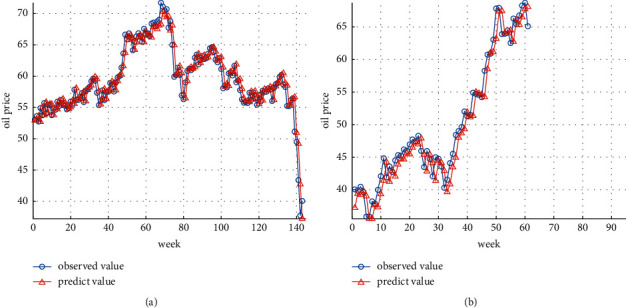
(a) GA-SVR prediction results of training dataset. (b) GA-SVR prediction results of test dataset.

Combining the parameter optimization results of GA, this paper uses the parameter optimization results of the penalty parameter *C*, the insensitive loss function *ε*, and the RBF kernel function parameters *γ* for establishing an SVR model to predict the oil future price time series. The prediction results of training dataset and test dataset are shown in Figures 3(a) and 3(b), respectively.

Figures 3(a) and 3(b) indicate the ability of the GA-SVR model to accurately predict the overall effect of oil future price index and can accurately depict the relationship between nonlinear fluctuations and linear trends. For further judging the prediction effect of the GA-SVR model, this paper further calculates the corresponding MSE, RMSE, MAE, and MAPE.

In later, we compare prediction evaluation indexs of GA-SVR model with prediction evaluation indexs of GA-SVR-GRNN model based on prediction evaluation indexs in Table 1.

### 3.4. Residual Prediction Based on Generalized Neural Network Model (GRNN)

In the environment of Matlab R2018b, this paper further establishes a GRNN model to predict the prediction residuals of GA-SVR and then forms a GA-SVR-GRNN hybrid deep learning algorithm. The specific steps are detailed in the following sections.

#### 3.4.1. Visualization of Residuals

**Figure 4:**
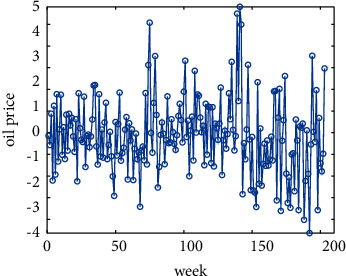
GA-SVR model residual graph.

From Figure 4, we found that the residual sequence of the GA-SVR model fluctuates around the value of 0, which has strong volatility.

#### 3.4.2. GRNN Optimal Smoothing Factor Selection

**Figure 5:**
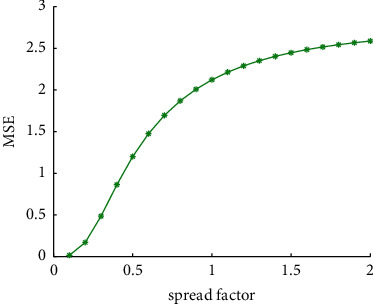
Optimal smoothness factor iteration process.

We follow existing literature in the paper. The initial smoothing factor is set to 0.1, and one unit amount (0.1) is added each time. The spread factor is set as the optimal value in the case that the above mean square error is the smallest [30]. Now plot the MSE of the points to be estimated of different smoothing factors between all the predicted values and the measured values of the sample (Figure 5). According to Figure 5, after checking the output results, we found that when the smoothing factor is 0.1, the error is the smallest in both the approximation performance and the prediction performance. With the increase of the smoothing factor, the error is also increasing. Therefore, this paper chooses the smoothing factor as 0.1.

#### 3.4.3. Residual Prediction of the GRNN Model

**Figure 6:**
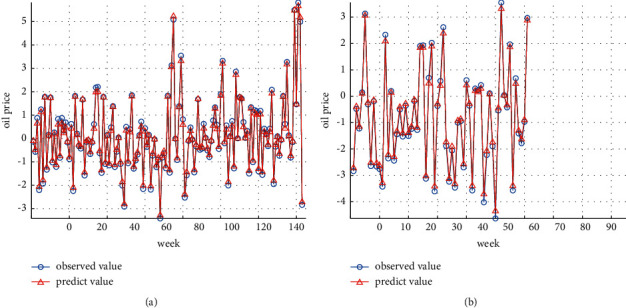
(a) GRNN model prediction results of training dataset. (b) GRNN model prediction results of test dataset.

In this paper, we select a GRNN network model of which the smoothing factor is 0.1 to train the residuals and output the residual prediction value of the GRNN model. On this basement, we compare the residual prediction value predicted by GRNN with the actual value (Figure 6). Figures 6(a) and 6(b) display the prediction results of the training dataset and the test dataset, respectively.

From Figures 6(a) and 6(b), the GRNN model displays an accurate prediction effect. It can capture the nonlinear change characteristics of the GA-SVR residual sequence, indicating that the GRNN model established in this paper has strong robustness.

### 3.5. Forecast Based on the GA-SVR-GRNN Hybrid Model

**Figure 7:**
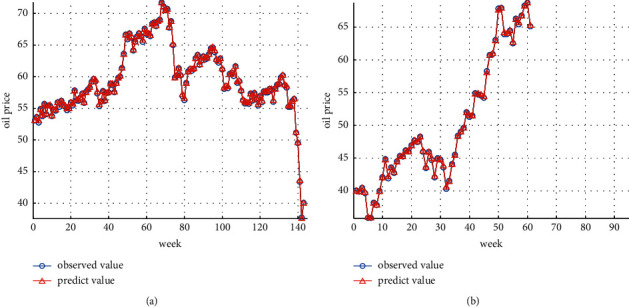
(a) GA-SVR-GRNN hybrid deep learning algorithm prediction results of the training dataset. (b) GA-SVR-GRNN hybrid deep learning algorithm prediction results of the test dataset.

This article sums up the oil future price time series predicted by GA-SVR and the residual error series predicted by GRNN to obtain the oil future price forecast value of the GA-SVR-GRNN combined model, draws the relevant time series diagram as Figures 7(a) and 7(b), and calculates the corresponding statistical evaluation index as summarized in Table 1. The prediction results of training set are shown in Figure 7(a). The prediction results of test set are shown in Figure 7(b).

For more deeply evaluating the pros and cons of the GA-SVR-GRNN hybrid deep learning algorithm for the oil future price, the paper calculates the relevant prediction and evaluation indicators and summarizes them as shown in Table 2.

By comparing Tables 1 and 2, we discovered that the GA-SVR-GRNN hybrid deep learning algorithm's evaluation indicators are substantially smaller than the GA-SVR single model, indicating that the combination model presents a remarkably stronger oil future price prediction accuracy compared with the single model. The model's prediction result is reliable, can be used to make informed decisions, and is extremely practical.

## 4. Further Comparative Analysis of Various Methods

### 4.1. PSO-SVR Single Model Comparison Test

The PSO-SVR model enjoys a wide application in energies forecasting field [44]. This paper uses the PSO algorithm for optimizing the parameters of the SVR and establishing a PSO-SVR model for the prediction of the oil future price.

#### 4.1.1. Particle Swarm Optimization (PSO) Algorithm Optimization Parameters

**Figure 8:**
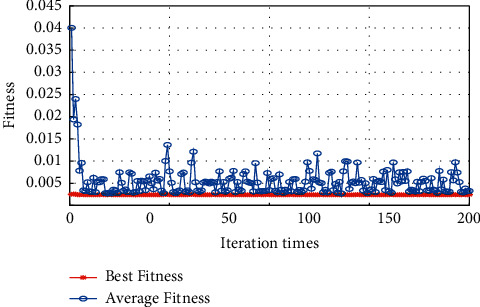
PSO optimization parameter fitness curve.

In the environment of Matlab R2018a, we use particle swarm optimization (PSO) for optimizing *C*, *ε*, and *γ* in the SVR model. The evolutionary algebra is set to 200 generations, the population size is 20, and the fitness function is selected as MSE for five-fold cross-validation. The results of the PSO optimization are shown in Figure 8. Finally, based on the PSO, the optimal penalty parameter *C* determined is 14.6549, the parameter *γ* of the RBF kernel function is 100, and the parameter *ε* of the insensitive loss function is 0.01.

From Figure 8, we found that the MSE value corresponding to the final optimization result of C, *ε*, and *γ* of the RBF kernel function are all less than 0.005, indicating the optimization parameter result of the PSO has strong adaptability.

#### 4.1.2. PSO-SVR Model Prediction

**Figure 9:**
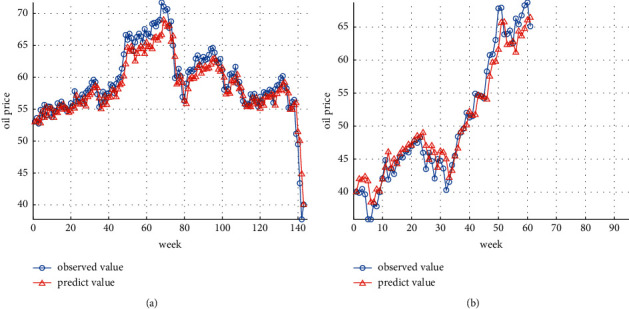
(a) PSO-SVR prediction results of the training dataset. (b) PSO-SVR prediction results of the test dataset.

According to the parameter optimization results of the PSO, this paper uses the parameter optimization results of the penalty parameter *C*, the insensitive loss function *ε*, and the RBF kernel function parameters *γ* for establishing an SVR model to predict the oil future price time series. Figures 9(a) and 9(b) display the prediction results, with the former showing the results of training dataset and the latter showing those of the test dataset.

For further judging the prediction effect of the PSO-SVR model over oil future price time series, we further calculate the MSE, RMSE, MAE, and MAPE.

Observing the PSO-SVR prediction effect evaluation indicators in Table 3, we found that the MSE, RMSE, MAE, and MAPE are all higher than those of GA-SVR, which is also significantly higher than the corresponding indicators of the GA-SVR-GRNN hybrid deep learning algorithm. It shows that the GA-SVR-GRNN hybrid deep learning algorithm exhibits an obviously better prediction effect relative to the GA-SVR model and the PSO-SVR model.

### 4.2. GRNN Single Model Comparison Experiment

**Figure 10:**
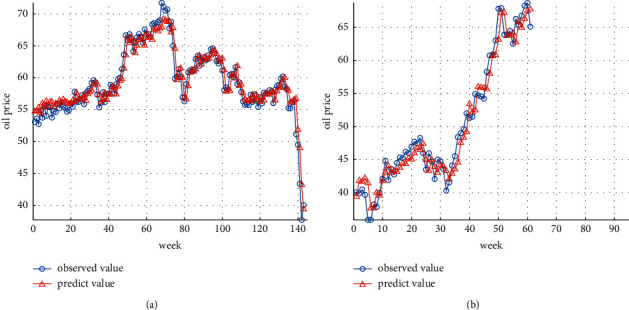
(a) PSO-SVR prediction results of the training dataset. (b) PSO-SVR prediction results of the test dataset.

In the environment of MATLAB R2018a, GRNN is established for the prediction over the time series of oil future prices. First, we need to figure out the optimal smoothing factor of the GRNN model. Based on the foregoing method, we select the GRNN network model of which the smoothing factor is 0.1 for the prediction over the time series of oil future prices and compare it with the actual results (Figures 10(a) and 10(b)). Figures 10(a) and 10(b) give the prediction results of training dataset and the test dataset, respectively.

For further judging the prediction effect of the GRNN model over oil future price time series, we further calculate the MSE, RMSE, MAE, and MAPE.

Observing the GRNN prediction effect evaluation indicators in Table 4, we found that GRNN exhibits higher MSE, RMSE, MAE, and MAPE relative to the GA-SVR-GRNN hybrid deep learning algorithm. It shows that the GA-SVR-GRNN hybrid deep learning algorithm in this paper has an obviously better prediction effect relative to the GRNN model.

## 5. Conclusions

This article selects the North Sea Brent oil futures settlement future price week from June 2, 2017, to May 21, 2021. Based on the data, we established the GA-SVR-GRNN hybrid deep learning algorithm for forecasting oil future price. The main research conclusions of this article are as follows:

First, through empirical research on the weekly data of Brent oil futures settlement future price in the North Sea, this article predicts the trend of oil future prices with relative accuracy. According to the simulation, the GA-SVR-GRNN hybrid deep learning algorithm achieves lower MSE, RMSE, MAE, and MAPE relative to the GRNN, GA-SVR, and PSO-SVR models, indicating that the proposed GA-SVR-GRNN hybrid deep learning algorithm can fully reveal the prediction advantages of the GA-SVR and GRNN models in the nonlinear space and is a more accurate and effective method for oil future price forecasting. The research findings can help some related organizations and experts to predict the oil future prices reasonably, which can remarkably optimize the production structure and decrease the risk caused by oil future price fluctuations.

Second, this article found that the GA-SVR-GRNN hybrid deep learning algorithm exhibits an obviously better effect relative to the GA-SVR model, PSO-SVR model, and GRNN model, which shows that reasonable hybrid prediction is more accurate and effective than single model prediction. The oil future price time series actually present complex nonlinear structural and chaotic characteristics, and it is difficult to fully capture the complex information behind the oil future price time series based on a single model, which results in the forecasting effect of a single model is not ideal sometimes. Therefore, this research took advantages of GA-SVR and GRNN models in the nonlinear forecasting space and realizes the prediction of oil future price more accurately.

Third, the hybrid model of this article has undergone comparative experiments. It is found that the prediction results of MSE, RMSE, MAPE, and MAE are significantly smaller than those of other comparative experiments, and the prediction results are more accurate. The reason is that the GA-SVR-GRNN hybrid deep learning algorithm is capable of comprehensively combining the advantages exhibited by GA-SVR and GRNN models. At the same time, the actual prediction results are consistent with reality, which also confirms that the model is effective and practical.

## Figures and Tables

**Table 1:** GA-SVR model prediction evaluation index.

Training dataset				
Model	MSE	RMSE	MAE	MAPE
GA-SVR	2.5227	1.5883	1.1632	2.0392

Test dataset				
Model	MSE	RMSE	MAE	MAPE
GA-SVR	4.3779	2.0924	1.6994	3.4915

**Table 2:** GA-SVR-GRNN model prediction evaluation index.

Training dataset				
Model	MSE	RMSE	MAE	MAPE
GA-SVR-GRNN	0.0129	0.1136	0.0961	0.1643

Test dataset				
Model	MSE	RMSE	MAE	MAPE
GA-SVR-GRNN	0.0185	0.1359	0.1221	0.2481

**Table 3:** PSO-SVR model prediction evaluation index.

Training dataset				
Model	MSE	RMSE	MAE	MAPE
PSO-SVR	3.7967	1.9485	1.5351	2.6009

Test dataset				
Model	MSE	RMSE	MAE	MAPE
PSO-SVR	5.4907	2.3432	1.7428	3.5256

**Table 4:** GRNN model prediction evaluation index.

Training dataset				
Model	MSE	RMSE	MAE	MAPE
GRNN	2.5408	1.5940	1.1875	2.0653

Test dataset				
Model	MSE	RMSE	MAE	MAPE
GRNN	3.9397	1.9849	1.6646	3.4035

## Data Availability

All data that are generated or analyzed in the study are included in this article.
